# Prevalence of intrinsic capacity decline among community-dwelling older adults: a systematic review and meta-analysis

**DOI:** 10.1007/s40520-024-02816-5

**Published:** 2024-08-01

**Authors:** Xia Cao, Xuanzi Yi, Hui Chen, Yusheng Tian, Sihong Li, Jiansong Zhou

**Affiliations:** 1 Health Management Center, The Third Xiangya Hospital of Central South University, No. 138, Tongzipo Road, Changsha, 410013 China; 2 Department of General Practice, The Third Xiangya Hospital of Central South University, No. 138, Tongzipo Road, Changsha, 410013 China; 3 National Clinical Research Center for Mental Disorders, Department of Psychiatry, The Second Xiangya Hospital of Central South University, Changsha, 410008 China

**Keywords:** Intrinsic capacity decline, Old adults, Prevalence, Meta-analysis, Systematic review

## Abstract

**Background:**

The concept of intrinsic capacity (IC) was introduced to define healthy aging and active aging based on functional capacity, yet there is limited understanding of the risk of IC decline at a population level.

**Aims:**

To consolidate existing evidence for rates of IC decline and risk factors among community-dwelling adults 60 years or older.

**Methods:**

According to the PRISMA guidelines, the literature search was independently conducted by two researchers in 8 databases from inception to January 2024 without language restrictions using combinations of free words and subject words. Qualities of included studies were assessed using Joanna Briggs Institute’s (JBI’s) critical appraisal checklist for prevalence studies. To pool the data, a random-effect meta-analysis was performed, followed by subgroup analysis and sensitivity analysis. All analyses were performed by Stata14.0.

**Results:**

From 1594 records, 15 studies were extracted with 33,070 participants for meta-analysis. The pooled prevalence of IC decline in community settings was 67.8% (95% CI: 57.0-78.5%; *P* < 0.001). The prevalence of IC decline in China (66.0%; 95% CI: 53.2-78.9%) was found to be slightly lower than in other countries/regions (73.0%; 95% CI: 59.8-86.3%); however, this difference was not statistically significant. Other subgroup analyses revealed no statistically significant differences in prevalence. Age, hypertension, diabetes, gender, education level, living status, smoking, regular exercise, marital status, and osteoarthritis are associated with IC decline.

**Conclusion:**

More than two-thirds of older adults in the community are affected by IC decline, and age, hypertension, diabetes, female sex, low education level, living alone, smoking, irregular exercise, unmarried, and osteoarthritis are all risk factors for IC decline.

**Supplementary Information:**

The online version contains supplementary material available at 10.1007/s40520-024-02816-5.

## Introduction

The process of aging is associated with a heightened susceptibility to physical and cognitive decline, potentially resulting in disability and a reduction in independence. Concurrently, variations in cognitive and physical abilities among individuals become more pronounced as the aging process advances and accelerates [1]. The World Health Organization (WHO) defined healthy aging as the process of establishing and maintaining functional capacity that enables well-being in older adulthood and advocated in its World Report on Ageing and Health to change the focus from “disease” to “capacity” in older adults [2]. The introduction of the concept of intrinsic capacity (IC) in this report aims to comprehensively assess the overall functioning of older adults, while also laying the groundwork for further research on IC assessment and the implementation of interventions to improve IC.

To integrate the concept of intrinsic capacity (IC) within a clinical setting, the World Health Organization (WHO) has delineated five essential domains of IC (namely locomotion, vitality, cognitive, psychological, and sensory) and introduced the Integrated Care for Older People (ICOPE) framework as a novel strategy aimed at enhancing IC to promote overall health and well-being [3]. Despite early studies supporting the validity of the WHO Healthy Ageing framework built around the concept of IC, there was variation in the measurement process or tools used for assessing IC domains and there was no clear criterion for quantifying (or scoring) IC as a global measure [4]. A recent review found that the most commonly reported measurement tool in IC intervention studies was an IC Z-score, calculated from four domains: locomotor, vitality, cognitive, and psychological [5]. These conceptual advances and accompanying transformative research paradigms offer great potential for scientists and scholars to understand the determinants of healthy aging and possible opportunities for innovative interventions [6].

It has been demonstrated that the five domains of capacity can be aggregated into an overall construct of IC, which predicts functional outcomes regardless of chronological age or chronic diseases [7, 8]. Furthermore, IC has been associated with polypharmacy [9], poor self-rated health [10], frailty [11], falls [12], and long-term nursing home residency [13]. There is also increasing evidence that IC decline is associated with mortality and other negative health outcomes in older adults, although the majority of these studies did not have long follow-up times [12, 14–16]. In recent years, in the context of healthy aging and the global COVID-19 pandemic, IC-related literature is increasing year by year [17, 18]. Given that frailty is prevalent among older adults, the IC impairment is therefore very important and needs more attention.

The prevalence of IC decline ranges from 55 to 77% among older adults in different countries (Singapore, France, and China) and settings (community or hospital) [19–21]. Recently, a meta-analysis reported the pooled prevalence of IC impairment was 55.0% among older adults in different global settings across several international databases [22]. Therefore, the prevalence of IC decline varies across countries and settings and can also differ based on the method of assessment. Since IC is a concept that has emerged recently, warranting a thorough and critical examination of the existing literature. As global interest in healthy aging continues to increase, a more comprehensive understanding of the prevalence of IC decline and risk factors could contribute to advancing discussions on the preservation of functional capacity in older populations. Considering this gap, this study aimed to systematically assess the prevalence and risk factors of IC decline among community-dwelling adults.

## Methods

### Protocol

This review followed PRISMA guidelines for meta-analysis [23]. All articles were found online, so institutional review board approval was not needed. The protocol is registered in PROSPERO (CRD42023488594).

### Search strategy

We searched PubMed, Embase, The Cochrane Library, Web of Science, China Knowledge Resource Integrated Database (CNKI), Wanfang Database, Chinese Biomedical Database (CBM), and China Science and Technology Journal Database (CQVIP) for studies from inception to January 2024 without language restrictions. The search terms included MESH terms and keywords. The retrieval keywords included intrinsic capacity, ICOPE, aged, elder, et al. Keywords in the same category were combined with “OR,” whereas those in different categories were combined with “AND.” The retrieval adopts the combination of subject words and free words. The search strategies were adjusted depending on each database. The detailed search strategy in English databases is shown in Supplementary Tables 1–4. Moreover, we screened the references of identified articles, reviews, or other relevant documents.

### Inclusion and exclusion criteria

The inclusion criteria were as follows: (1) Community-dwelling older adults aged 60 years or above; (2) The study participants were assessed for IC based on ICOPE. IC decline was operationally defined as a reduction in scores on any of the dimensions; (3) cross-sectional studies and cohort studies, with no language restrictions; and (4) studies that reported the prevalence of IC decline, using a clear definition of IC decline. The exclusion criteria were as follows: (1) reviews, letters, comments, or conference abstracts; (2) data on the prevalence of IC decline were not available or were insufficient to calculate the prevalence of IC decline; (3) research on older adults living in institutions, hospital or nursing homes; (4) the publication was in a language other than English or Chinese.

### Study selection and data extraction

Two investigators (X.C. and H.C.) independently screened records based on the title, abstract, and full texts, after deleting duplicated articles using Endnote 21. Where disagreements arose, they would be settled through consensus with a third reviewer. Data extraction was carried out by three authors (X.Z.Y., Y.S.T., and H.S.L.), who checked one another’s results and discussed any disagreements. The following information was collected from individual articles: study details (authors, year of publication, country, setting, and study design), participant characteristics (sample size, age, and case of female/male participants), IC measurement method, prevalence of IC decline, study outcome definition, and selection method. Data on prevalence stratified by gender or age were collected, where available. In cohort studies, we collected baseline data on IC decline when necessary.

### Assessment of study quality

Two investigators (X.C. and H.C.) evaluated each included study for methodological quality using the Joanna Briggs Institute’s critical appraisal tool for prevalence studies [24]. The checklist consists of nine criteria, and a study was ineligible if fewer than five of the criteria were met.

### Statistical analysis

In this study, the prevalence of IC decline was either derived directly from the article or calculated based on the available data. Heterogeneity across studies was estimated using Cochran’s Q test and *I*^*2*^ statistic. A Cochran Q statistic *P* < 0.05 or *I*^*2*^ > 50% indicated the presence of statistically significant heterogeneity. There was a significant degree of heterogeneity across the studies (*I*^*2*^ = 99.86%) because they varied in inclusion criteria, study design, and IC assessment criteria. Therefore, a random-effects model was used to calculate the pooled effect size. Publication bias was studied using Funnel plots, the Egger method, and trim-and-fill analysis [25, 26]. We also performed a broad subgroup analysis based on study design, country, gender, and age. Finally, sensitivity analysis was conducted to evaluate the effect of the included study on the prevalence rate of IC decline and the robustness of the results from the meta-analysis. Statistical analysis was performed using Stata14.0 (StataCorp). Two-tailed *P* < 0.05 was considered statistically significant.

## Results

### Selection process

**Fig. 1:**
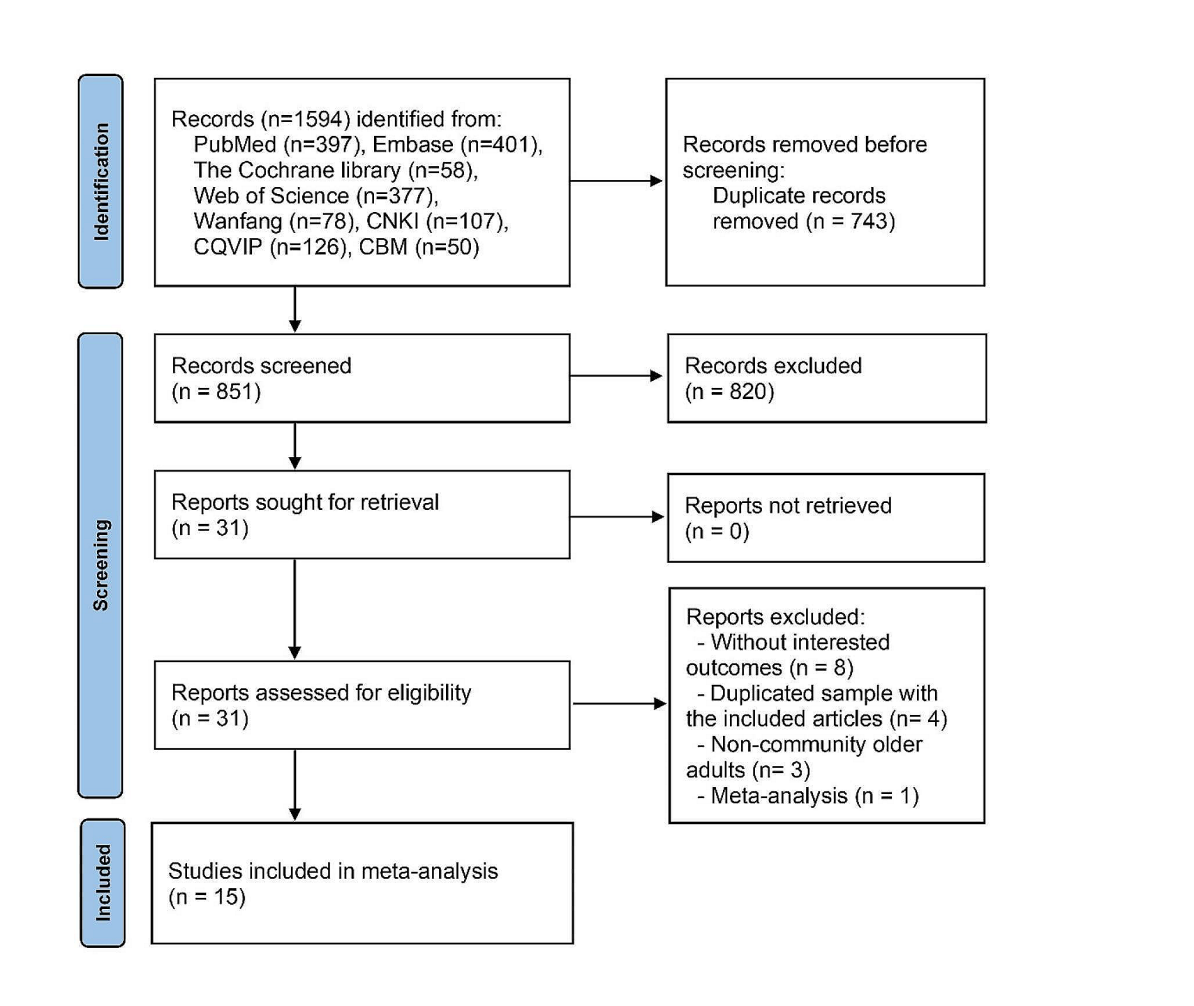
Flow diagram of studies selection

Figure 1 illustrates a selection process with the number of studies at each stage. At first, 1,594 articles were identified. After removing duplicates, 851 were screened. Of them, 820 were excluded after the review of titles and abstracts, thus leaving 31 articles for full-text screening. Of these, 8 records without interested outcomes, 4 records with duplicated samples, and 4 studies did not meet the inclusion criteria. This gave a total of 15 studies for review and analysis.

### Study characteristics

The characteristics of the included studies are summarized in Table 1. Fifteen studies (*n* = 33,070) from 5 countries were obtained. Most of the studies were from Asia, with 11 from China [10, 21, 27–35], 3 from other Asian countries [36–38], and 1 from Europe [39]. Studies were published between 2021 and 2023, with sample sizes ranging from 196 to 10,007 participants. The mean or median age ranged from 66.5 to 84.0 years, and 3 studies did not provide the detailed mean or median age for the total sample [32, 38]. Most studies were cross-sectional (10), with 6 cohort studies, from which we used data from the baseline assessment (the study of Zhang et al. included 2 independent cohorts [38]). The screening of IC decline was performed based on ICOPE in all the included studies. However, the assessment tools used were not consistent. Supplementary Fig. 1 shows the frequency of the measurement tools related to IC and its five domains reported in 15 included IC decline prevalence studies.

**Table 1:** Characteristics of 15 included studies in this meta-analysis

Study	Country	Design	Age, years	*N*, M/F	IC decline	Prevalence (%)
Cheng, YC 2021	China	CSS	73 ± 6	457, 205/252	78	17.07%
Jiang, X 2023	China	CSS	71.00 (68.0-76.8)	968, 402/566	704	72.73%
Leung, AYM 2022	China	CSS	76.73 ± 7.25	304, 57/237	221	72.70%
Lin, S 2023	China	CSS	71 (66–77)	1972, 794/1178	1937	98.23%
Liu, S 2021	China	CS	83.7 ± 4.4	196, 80/116	133	67.86%
Lu, F 2023	China	CS	84.0 ± 4.4	228, 97/131	167	73.25%
Ma, L 2021	China	CSS	60–98	5823, 2518/3305	2506	43.04%
Rarajam Rao, A 2023	India	CSS	66.5 (63–73)	1000, 371/629	843	84.30%
Rojano I. Luque X 2023	Spain	CSS	76.7 (74.1–79.9)	207, 81/126	164	79.23%
Saiyare, X 2023	China	CSS	71.98 ± 8.20	1072, 530/542	787	73.41%
Tay, L 2023	Singapore	CS	67.6 ± 6.8	809, 197/612	596	73.67%
Yu, R 2022	China	CSS	75.7 ± 7.9	10,007, 2086/7921	8492	85.34% ^a^
Zhang, S 2023	China	CS	60.0-86.5	1358, 462/896	506	37.26%
	Japan	CS	60.0-96.7	794, 407/387	436	54.91%
Zhao, J 2021	China	CS	74.2 ± 5.5	7298, 2851/4447	4709	64.52%
Zhao, Y 2023	China	CSS	72.5 ± 7.3	577, 365/212	502	87.00%

CSS, Cross-sectional study; CS, Cohort study; IC, intrinsic capacity; M, male; F, female

^a^, data of IC assessment was 56 missings, remaining 9951 participants

### Methodological quality

The results of the quality assessment are presented in Supplementary Table 5. A score of 6–7 for cohort studies and 5–7 for cross-sectional studies. Overall, the methodological quality of the included studies was high, and the risk of bias was low.

### Prevalence of IC decline

**Fig. 2:**
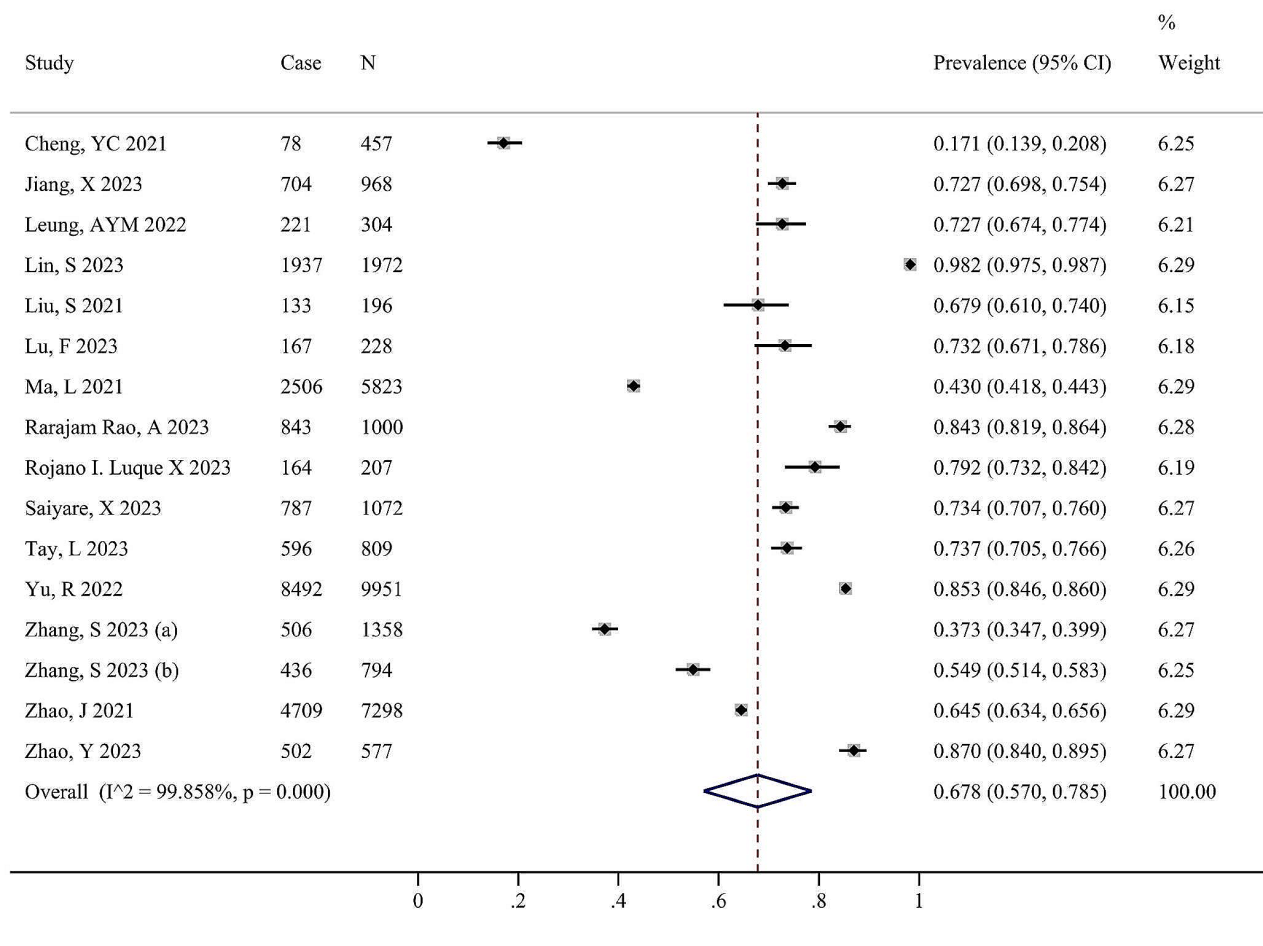
Meta-analysis for the prevalence of intrinsic capacity decline among community-dwelling older adults

The prevalence of IC decline in individual studies ranged from 17.1 to 98.2%. The pooled prevalence of so IC decline was 67.8% (95% CI: 57.0-78.5%, I^2^ 99.86%, *P* < 0.001) (Fig. 2).

### Subgroup analyses

**Fig. 3:**
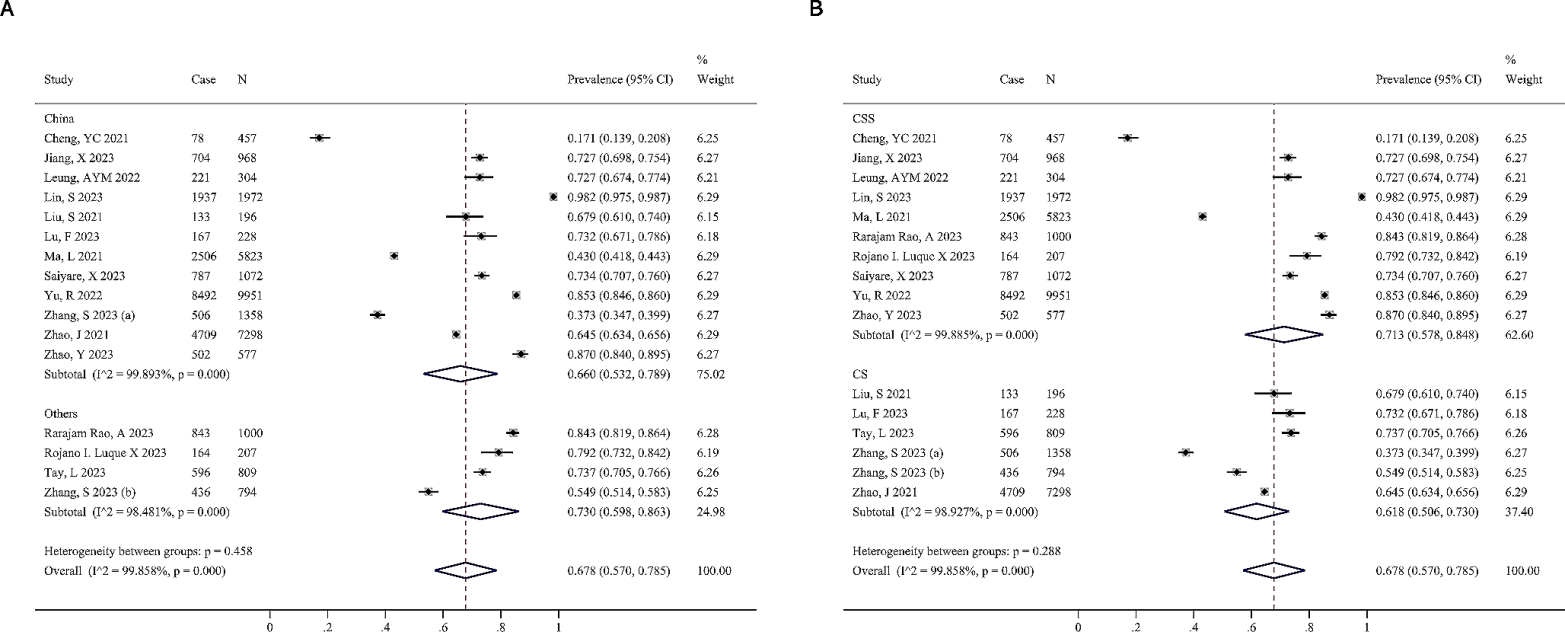
(**A**) Subgroup analyses by country. (**B**) Subgroup analyses by study types

**Fig. 4:**
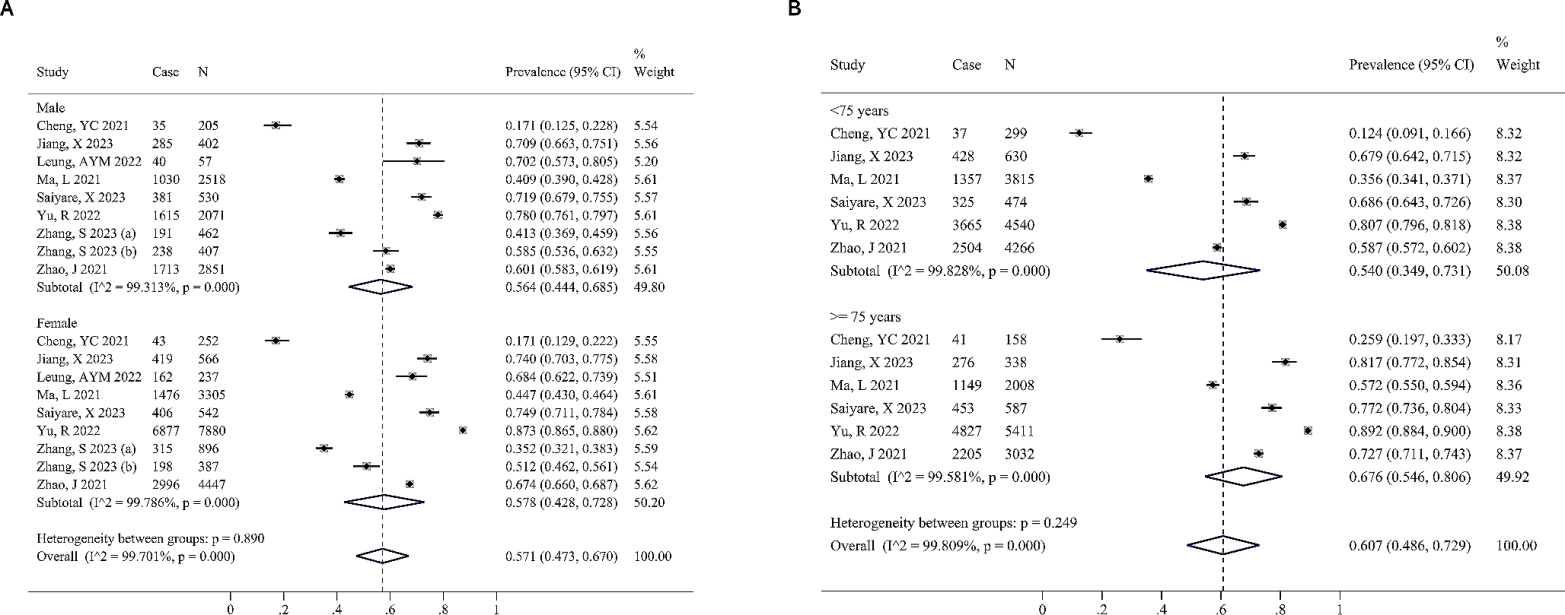
(**A**) Subgroup analyses by gender. (**B**) Subgroup analyses by age

The China subgroup had a slightly lower prevalence of IC decline compared to the Others subgroup, 0.660 (0.532, 0.789) vs. 0.730 (0.598, 0.863), but the difference was not statistically significant (*P* = 0.458, Fig. 3A). Furthermore, the subgroup analysis based on study type revealed that the disparity between the combined outcomes of cohort study (CS) subgroups (PR = 0.618, 95%CI = 0.506–0.730) and cross-sectional study (CSS) subgroups (PR = 0.713, 95% CI = 0.578–0.848) was not statistically significant (*P* = 0.288, Fig. 3B). Several of the studies included in the analysis documented the prevalence of IC decline among males, females, and various age groups. There was a small difference between males (PR = 0.564, 95%CI = 0.444–0.685) and females (PR = 0.578, 95%CI = 0.428–0.728) with a *P* value of 0.890 (Fig. 4A). The study found that individuals under 75 years of age exhibited a lower prevalence of IC decline (PR = 0.540, 95%CI = 0.349–0.731) compared to those aged 75 years and older (PR = 0.676, 95%CI = 0.546–0.806), but the difference was not statistically significant (*P* = 0.249, Fig. 4B). High within-group heterogeneity (I^2^ > 95%, *P* < 0.001) remained in the subgroup analysis, indicating that factors such as study type, region, gender, and age did not significantly affect heterogeneity.

### Risk factors

The systematic review encompassed 15 articles that collectively identified ten risk factors linked to IC decline among older adults residing in community settings, including age, hypertension, diabetes, female sex, low education level, living alone, smoking, irregular exercise, unmarried, and osteoarthritis, which were used for pooled analysis (Table 2).

**Table 2:** Combined risk factors for intrinsic capacity decline

Risk Factors	OR (95%CI)	*P*_A_	Heterogeneity test	
			*P*	I^2^ (%)
Age ( > = 75 vs. <75 years)	1.92 (1.78, 2.06)	< 0.001	0.516	0.0
Hypertension (Yes vs. No)	1.46 (1.24, 1.72)	< 0.001	0.370	4.6
Diabetes (Yes vs. No)	1.18 (0.94, 1.49)	0160	0.506	0.0
Gender (Female vs. Male)	1.02 (0.80, 1.29)	0.890	< 0.001	92.8
Education ( < = 9 vs. >9 years)	1.99 (1.83, 2.17)	< 0.001	0.547	0.0
Living status (Living alone vs. With family)	1.28 (0.96, 1.71)	0.090	0.071	53.7
Smoking (Yes vs. No)	1.58 (1.16, 2.15)	0.003	0.139	49.3
Regular exercise (No vs. Yes)	1.40 (1.17, 1.68)	< 0.001	0.208	37.0
Marital status (Unmarried vs. Married)	1.58 (1.45, 1.72)	< 0.001	0.131	39.1
Osteoarthritis (Yes vs. No)	1.42 (1.07, 1.89)	0.016	0.051	66.5

*P*_*A*_: *P* value for test of the association

### Publication bias and sensitivity analysis

The symmetry of the funnel plots was not obvious (Supplementary Fig. 2A), and the results of Begg’s test suggested a possible publication bias (*P* = 0.046). We further applied a trim and filling analysis to evaluate the impact of publication bias on the results, and the trim and fill estimate (PR = 0.642, 95%CI = 0.496–0.788) did not significantly alter the results (Supplementary Fig. 2B). After eliminating the included studies one by one and combining the rest using sensitivity analysis, there were no significant differences between the combined effect value and the total combined value, suggesting that the study results were consistent (Supplementary Fig. 3). Stata’s “hatred” procedures were used to identify studies that had a greater impact on heterogeneity. The results showed that the eight articles (including Lin, S 2023, Yu, R 2022, Ma, L 2021, Cheng, YC 2021, Zhang, S 2023, Zhao, J 2021, Zhao, Y 2023, Rarajam Rao, A 2023 ) have a great impact on heterogeneity. After exclusion, the heterogeneity test results of the remaining 7 articles were as follows: *I*^2^ = 18.4%, *P* = 0.289 (Supplementary Fig. 4). The combined result was PR (95%*CI*) = 0.734 (0.717, 0.750), which showed larger effect size and higher statistical accuracy than the original combined result. However, the difference between the two was not significant (*P* = 0.313), and the results of meta-analysis were robust.

## Discussion

The meta-analysis showed that the pooled prevalence of IC decline among community-dwelling older adults was 67.8% (95% CI: 57.0-78.5%). The subgroup analyses suggest that country and age may influence the prevalence of IC decline in older adults, although the differences were not statistically significant. The finding implies that more than two-thirds of community-dwelling older adults experience IC declines. According to several of the studies included in the analysis, 23.0% participants were frail at baseline [30, 37] and IC decline was significantly correlated with an increased risk of frailty among in community- dwelling older adults [30, 32, 37]. Therefore, interventions focused on reducing the risk factors linked to IC decline in older adults may have substantial implications for promoting healthy aging. To the best of our knowledge, this is the first meta-analysis to summarize the evidence of IC decline prevalence and risk factors in community-dwelling older adults. Different from the above-mentioned meta-analysis [22], the present study excluded studies on institutionalized or hospitalized adults and nursing home residents because they are typically frail. Moreover, the database we searched includes both English and Chinese databases.

This study found differences in the prevalence of IC decline by country. The prevalence rate of IC decline in China (66.0%) was lower than for another country subgroup (73.0%). IC decline was lowest in Japan (54.9%), followed by Singapore (73.7%), Spain (79.2%), and India (84.3%) among community-dwelling older adults, excluding China. These variations may be attributed to disparities in sampling methods, and measurement instruments, as well as disparities in social structures, cultural norms, and economic conditions in each country [40]. Most studies in the review were from Asia, with only one from Europe. Additional research is needed in this area, particularly from countries not covered in this review. The subgroup analysis, stratified by study design, revealed that the pooled prevalence of IC decline was higher for the cross-sectional study design than for the cohort study type in this meta-analysis. The difference is likely due to variations in methodology, such as sample population and measurement tools used in cross-sectional and cohort studies (Supplementary Table 6). The majority of the cross-sectional studies originated in China, while two out of the six cohort studies were conducted in Singapore and Japan, which are countries with relatively low IC decline prevalence [37, 38].

Although the studies’ assessments of IC are based on the ICOPE framework, different assessment tools for domains of IC can also affect outcome judgments. For instance, in the case of hearing impairment, certain studies have employed objective measures [28, 38], whereas others have utilized self-reported formats [21, 29, 30, 38]. In addition, the most common assessment tool for the psychological domain was the Geriatric Depression Scale (GDS), which was used in 11 studies. Multiple versions of this scale were utilized in the pertinent research, such as GDS-15 [21, 30, 31, 34, 37], GDS-30 [32], and GDS-4 [29]. Although the WHO proposed the ICOPE screening tool to identify functional impairment, there is no consensus on the best tools for assessing IC in real-world studies. Subsequent examination indicated that all studies that did not utilize the Geriatric Depression Scale (GDS) or its abbreviated version, GDS-15, were carried out in China. This suggests the current challenges of establishing a standardized screening system for assessing IC decline in cross-cultural contexts and across ethnic characteristics.

The findings of this study indicate that the prevalence of IC decline was higher among individuals aged 75 years or older compared to those younger than 75 years. Surprisingly, no significant difference was found between them (*P* = 0.249). However, we believe that this difference between ages is objective. An earlier study discovered that older age increased the risk of IC decline [41]. Another study demonstrated a notable inverse relationship between advancing age and IC, suggesting that the IC decline may be a gradual phenomenon linked to the aging process [21]. Moreover, research examining the trajectories of IC in older adults indicates a gradual decline in intrinsic capacity with advancing age [42]. As individuals progress in age, they experience an accumulation of molecular and cellular damage, resulting in a deterioration of physical capabilities and an elevated susceptibility to illness, ultimately culminating in a decline in overall physiological functioning [43]. However, aging doesn’t necessarily mean impaired IC either. Jiang et al. found that the particular population of very old adults exhibited levels of IC comparable to those observed in younger older adults [21]. Researchers interpreted that older adults who volunteered for the study were more proactive in caring for their health, potentially leading to their higher intrinsic capacity.

To date, several interventions involving exercise, nutrition, cognitive training, and prehabilitation, have been assessed for their efficacy in delaying or reversing IC decline [44–47]. Most of these studies have shown that multi-domain interventions can help maintain IC in older adults, including those with declining self-care abilities [48]. Nevertheless, a recent systematic review has shown that essential strategies, such as the goal-setting approach and involving older adults in defining the strategy to maintain independence and activity, are crucial in the implementation of primary care interventions aimed at delaying or reversing IC decline [49]. This meta-analysis found that over 50% of individuals under 75 experienced IC decline, highlighting the importance of interventions to preserve IC in younger older adults.

The current study has several strengths. First, this meta-analysis is believed to be the initial investigation into the prevalence of IC decline among older individuals under the ICOPE framework, underscoring the importance of timely identification and intervention to alleviate its consequences. Second, a thorough examination was conducted through comprehensive analyses, encompassing various subgroup analyses, sensitivity analyses, and tests for publication bias, to confirm the robustness and validity of the study findings.

This study also had some limitations. First, the results should be taken with caution because significant heterogeneity was observed among the studies included in the analysis. However, it is important to note that heterogeneity is a common occurrence in meta-analyses of observational studies and does not necessarily undermine the validity of the results [50]. Based on the inclusion criteria, we decided to pool prevalence data across studies. Second, subgroup analysis was used to explore potential sources of heterogeneity, revealing significant differences in IC prevalence, but study type, region, gender, and age were not significant factors for heterogeneity. Third, the international sample exhibited an imbalance, with a notable disparity in the number of studies conducted in China compared to other countries. Due to the limited studies per country or region, it is impossible to determine how this may impact the results. Finally, different assessment tools and scoring criteria provide varying estimates, so caution should be taken when interpreting the meta-analysis results, especially when comparing across different measurement tools. However, one thing is consistent, the criterion of all studies for IC decline is that there is damage in any dimension.

## Conclusions

The community-dwelling older adults are prone to suffering from IC decline. The study found that it was influenced by country, sample population, and the assessment tools for IC. It is crucial to better understand the factors that increase the risk of IC decline. This will facilitate the creation of interventions designed to prevent the decline of IC or alleviate its negative impact on health. Additional research is necessary through prospective clinical trials or cohort studies to evaluate the most effective interventions and strategies for reducing the incidence of IC decline.

### Electronic supplementary material

Below is the link to the electronic supplementary material.

Supplementary Material 1

Supplementary Material 2

Supplementary Material 3

Supplementary Material 4

Supplementary Material 5

Supplementary Material 6

## Data Availability

No datasets were generated or analysed during the current study.
