# Supplementary figures and images for: Prevalence of intrinsic capacity decline among community-dwelling older adults: a systematic review and meta-analysis
Source: Aging Clin Exp Res. 2024 Aug 1;36(1):157. doi: 10.1007/s40520-024-02816-5 (PMC11294388; doi:10.1007/s40520-024-02816-5)

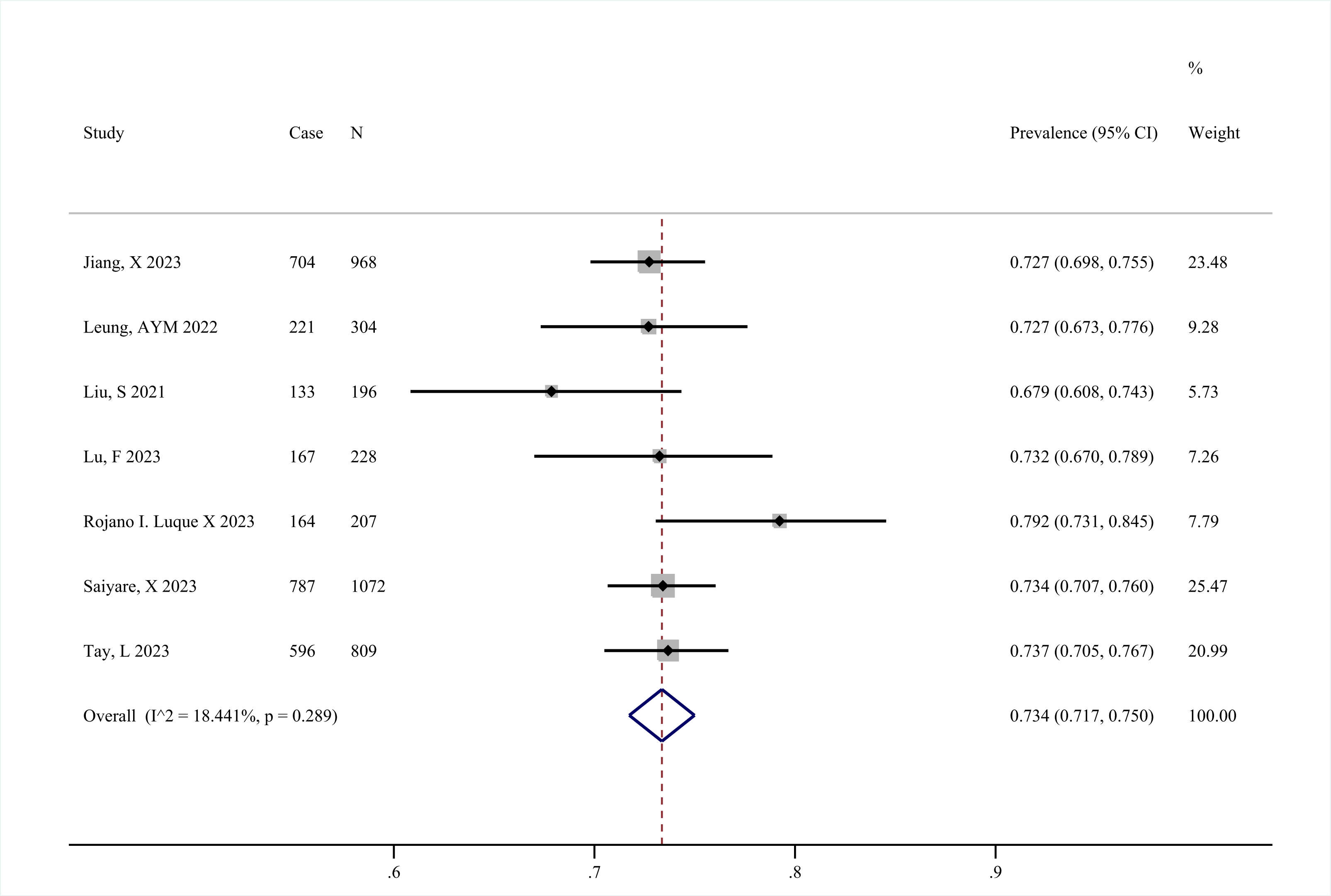

Supplement: Supplementary file 4 — Supplementary Material 4 [file 40520_2024_2816_MOESM4_ESM.tif]

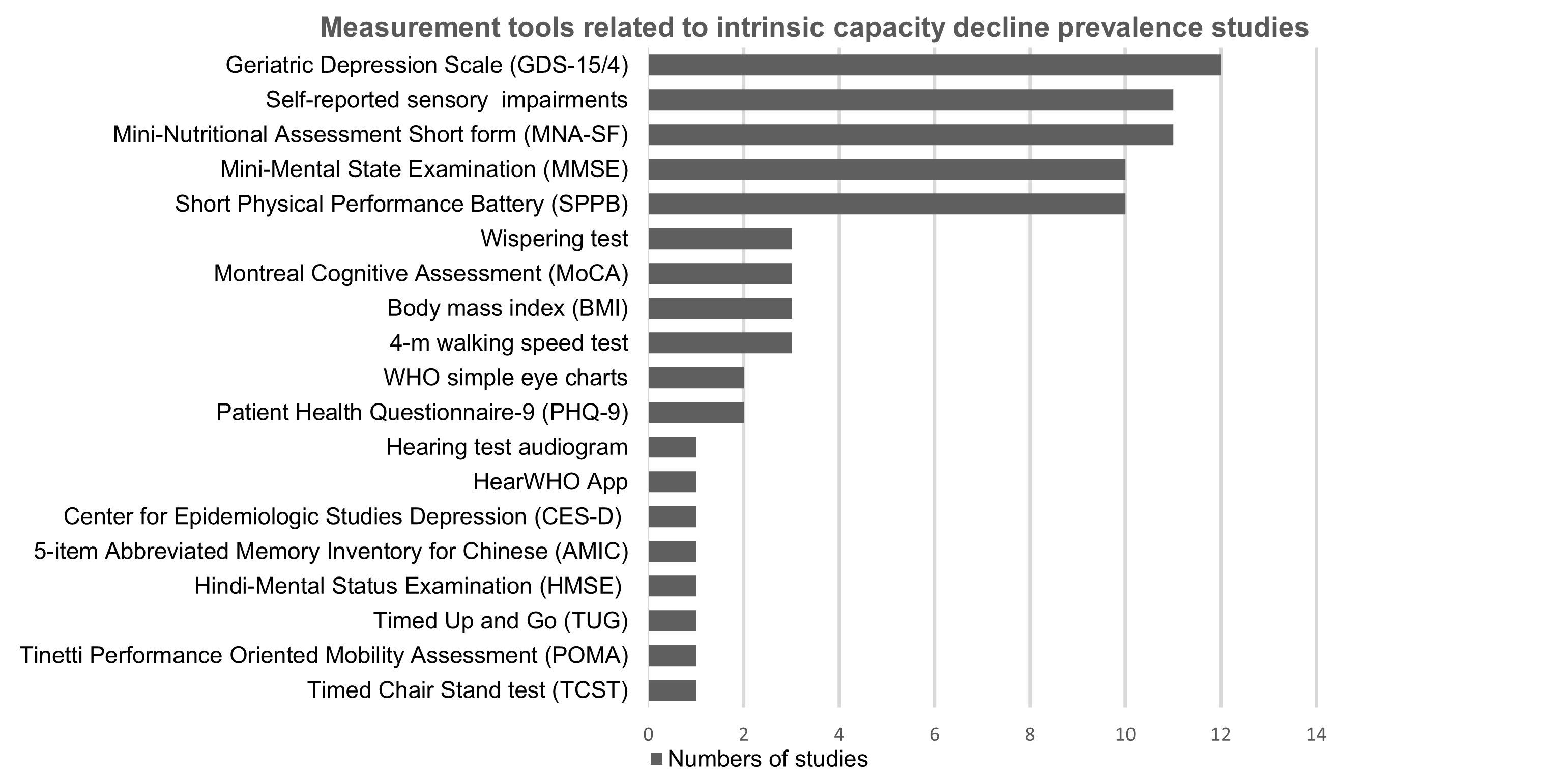

Supplement: Supplementary file 6 — Supplementary Material 6 [file 40520_2024_2816_MOESM6_ESM.tif]
