# Supplementary material for: Prevalence of intrinsic capacity decline among community-dwelling older adults: a systematic review and meta-analysis
Source: Aging Clin Exp Res. 2024 Aug 1;36(1):157. doi: 10.1007/s40520-024-02816-5 (PMC11294388; doi:10.1007/s40520-024-02816-5)
Supplement: Supplementary file 5 — Supplementary Material 5 [file 40520_2024_2816_MOESM5_ESM.docx]

**Supplementary Table 1 Retrieval strategies and results in PubMed**

| Search | Query | Items found |
| --- | --- | --- |
| #1 | "intrinsic capacity"[All Fields] | 1031 |
| #2 | "ICOPE"[All Fields] OR "Integrated Care for Older People"[All Fields] | 192 |
| #3 | #1 OR #2 | 1173 |
| #4 | ((("Aged"[Mesh]) OR ("Aging"[Mesh])) OR ((((((((aged[Title/Abstract]) OR (elder*[Title/Abstract])) OR (aging[Title/Abstract])) OR (senior*[Title/Abstract])) OR (old[Title/Abstract])) OR (geriatric[Title/Abstract])) OR (old people[Title/Abstract])) OR (old adults[Title/Abstract]))) | 5331141 |
| #5 | #3 AND #4 | 397 |

The retrieval time: 20240112

**Supplementary Table 2 Retrieval strategies and results in Embase.**

| Search | Query | Items found |
| --- | --- | --- |
| #1 | ('intrinsic capacity'/exp OR 'intrinsic capacity' OR icope OR 'integrated care for older people') | 1175 |
| #2 | ('aged' OR 'aging'/exp OR 'aging' OR 'aged'/exp OR aged OR elder* OR senior* OR 'geriatric'/exp OR geriatric OR 'old people' OR 'old adults') | 4746866 |
| #3 | #1 AND #2 | 401 |

**Supplementary Table 3 Retrieval strategies and results in Web of Science.**

| Search | Query | Items found |
| --- | --- | --- |
| #1 | ("intrinsic capacity") OR (ICOPE OR "Integrated Care for Older People") (All Fields) | 1151 |
| #2 | ("Aged" OR "Aging" OR aged OR elder* OR senior* OR geriatric OR "old people" OR "old adults") (All Fields) | 3742177 |
| #3 | #1 AND #2 | 377 |

**Supplementary Table 4 Retrieval strategies and results in The Cochrane library.**

| Search | Query | Items found |
| --- | --- | --- |
| #1 | ("intrinsic capacity" OR ICOPE OR "Integrated Care for Older People"):ti,ab,kw (Word variations have been searched) | 71 |
| #2 | MeSH descriptor: [Aged] in all MeSH products | 256396 |
| #3 | ("Aged" OR "Aging" OR aged OR elder* OR senior* OR geriatric OR "old people" OR "old adults"):ti,ab,kw (Word variations have been searched) | 889775 |
| #4 | #2 OR #3 | 889775 |
| #5 | #1 AND #4 | 58 |

**Supplementary Table 5 Quality assessment of the included studies based on the JBI tool.**

| **Study** | **Design** | **1** | **2** | **3** | **4** | **5** | **6** | **7** | **8** | **Total Score** |
| --- | --- | --- | --- | --- | --- | --- | --- | --- | --- | --- |
| Cheng, YC 2021 | CSS | Y | Y | Y | Y | Y | Y | Y | NA | 7 |
| Jiang, X 2023 | CSS | Y | Y | Y | Y | Y | Y | Y | NA | 7 |
| Leung, AYM 2022 | CSS | Y | Y | Y | Y | Y | Y | Y | NA | 7 |
| Lin, S 2023 | CSS | Y | Y | Y | N | N | Y | Y | NA | 5 |
| Liu, S 2021 | CS | Y | Y | Y | N | N | Y | Y | Y | 6 |
| Lu, F 2023 | CS | Y | Y | Y | N | N | Y | Y | Y | 6 |
| Ma, L 2021 | CSS | N | Y | Y | Y | Y | Y | Y | NA | 6 |
| Rarajam Rao, A 2023 | CSS | Y | Y | Y | Y | Y | Y | Y | NA | 7 |
| Rojano I. Luque X 2023 | CSS | Y | Y | Y | N | N | Y | Y | NA | 5 |
| Saiyare, X 2023 | CSS | Y | Y | Y | Y | Y | Y | Y | NA | 7 |
| Tay, L 2023 | CS | Y | Y | Y | Y | Y | Y | Y | N | 7 |
| Yu, R 2022 | CS | N | Y | Y | Y | Y | Y | Y | Y | 7 |
| Zhang, S 2023 | CS | Y | Y | Y | Y | Y | Y | Y | N | 7 |
| Zhao, J 2021 | CS | N | Y | Y | Y | Y | Y | Y | Y | 7 |
| Zhao, Y 2023 | CSS | Y | Y | Y | N | N | Y | Y | NA | 5 |

Note : Y, yes; N, no; NA, Not Applicable.

Criteria 1-7 for Assessing Study Quality for Cross-Sectional Studies Included in the Meta-analysis.

Criteria 1-8 for Assessing Study Quality for Cohort Studies Included in the Meta-analysis.

1. Were the criteria for inclusion in the sample clearly defined?

2. Were the study subjects and the setting described in detail?

3. Was the exposure measured in a valid and reliable way?

4. Were confounding factors specified?

5. Were strategies to deal with confounding factors stated?

6. Were the outcomes measured in a valid and reliable way?

7. Was appropriate statistical analysis used?

8. Was follow-up complete, and if not, were the reasons to loss to follow up described and explored?

**Supplementary Table 6 Summary of the definition or measurement tools used to assess the Intrinsic capacity domains in the included 15 studies**

| **Study** | **Design** | **Locomotor domain** | **Vitality domain** | **Cognitive domain** | **Psychological domain** | **Sensorial domain** |
| --- | --- | --- | --- | --- | --- | --- |
| Cheng, YC 2021 | CSS | Timed Chair Stand test (TCST) | Self-reported weight loss or appetite loss | Two questions on orientation in time and space or recall the three words should be remembered. | Participants being bothered feeling down, feeling depressed or hopeless, or having little interest or pleasure in doing things over two weeks. | Self-reported sensory impairments |
| Jiang, X 2023 | CSS | The short physical performance battery (SPPB) | Mini Nutritional Assessment Brief Form (MNA-SF) | Mini Mental State Examination (MMSE) | Geriatric Depression Scale (GDS-15) | Vision and hearing examinations; Self-reported sensory impairments |
| Leung, AYM 2022 | CSS | The short physical performance battery (SPPB) | Mini Nutritional Assessment Brief Form (MNA-SF) | Montreal Cognitive Assessment (MoCA) | Patient Health Questionnaire (PHQ‑9) | Whisper test, the Weber and Rinne test |
| Lin, S 2023 | CSS | 4-m walking speed test | Mini Nutritional Assessment Brief Form (MNA-SF) | Mini-cog | Geriatric Depression  Scale-4 (GDS-4) | Self-reported sensory impairments |
| Liu, S 2021 | CS | The short physical performance battery (SPPB) | Mini Nutritional Assessment Brief Form (MNA-SF) | Mini Mental State Examination (MMSE) | Geriatric Depression Scale (GDS-15) | Self-reported sensory impairments |
| Lu, F 2023 | CS | The short physical performance battery (SPPB) | Mini Nutritional Assessment Brief Form (MNA-SF) | Mini Mental State Examination (MMSE) | Geriatric Depression Scale (GDS-15) | Self-reported sensory impairments |
| Ma, L 2021 | CSS | The short physical performance battery (SPPB) | BMI | Mini Mental State Examination (MMSE) | Geriatric Depression Scale (GDS-15) | Self-reported sensory impairments |
| Rarajam Rao, A 2023 | CSS | Timed Up and Go (TUG) | BMI | Hindi-Mental Status Examination (HMSE) | Geriatric Depression Scale (GDS-15) | Wispering test |
| Rojano I. Luque X 2023 | CSS | The short physical performance battery (SPPB) | Mini Nutritional Assessment Brief Form (MNA-SF) | Mini Cognitive Examination (MEC) | Geriatric Depression Scale (GDS-5) | HearWHO App |
| Saiyare, X 2023 | CSS | The short physical performance battery (SPPB) | Mini Nutritional Assessment Brief Form (MNA-SF) | Mini Mental State Examination (MMSE) | Geriatric Depression Scale (GDS-15) | Self-reported sensory impairments |
| Tay, L 2023 | CS | The short physical performance battery (SPPB) | Mini Nutritional Assessment Brief Form (MNA-SF) | Mini Mental State Examination (MMSE) | Geriatric Depression Scale (GDS-15) | Self-reported sensory impairments |
| Yu, R 2022 | CSS | Self-reported the relevancy to mobility | Self-reported weight loss of ≥5% within the past 6 months | 5-item Abbreviated Memory Inventory for Chinese (AMIC) | Subjective well-being | Self-reported sensory impairments |
| Zhang, S 2023 | CS | 4-m walking speed test | Self-reported weight loss or appetite loss | Mini Mental State Examination (MMSE) | Epidemiologic Studies Depression (CES-D) | Self-reported sensory impairments/ Hearing test audiogram |
|  | CS | Timed Chair Stand test (TCST) | Self-reported weight loss or appetite loss | Montreal Cognitive Assessment (MoCA) | Epidemiologic Studies Depression (CES-D) | Self-reported sensory impairments |
| Zhao, J 2021 | CS | Tinetti score | Mini Nutritional Assessment Brief Form (MNA-SF) | Mini Mental State Examination (MMSE) | Geriatric Depression Scale (GDS-15) | Self-reported sensory impairments |
| Zhao, J 2023 | CSS | The short physical performance battery (SPPB) | Mini Nutritional Assessment Brief Form (MNA-SF) | Montreal Cognitive Assessment (MoCA) | Geriatric Depression Scale (GDS-15) | Self-reported sensory impairments |

CSS, Cross-sectional study; CS, Cohort study.
